# Comparative Accuracy of Preoperative Planning Technologies in Spinal Deformity Surgery: A Systematic Review

**DOI:** 10.7759/cureus.110221

**Published:** 2026-06-04

**Authors:** Meshal Jarebi, Mohammed S Alhomaidi, Salman H Ghzwani, Othman D Alamri, Abdulmalik M Almukhashi, Saud M Almousa, Abdulaziz S Almuhaisen, Mohammad F Aljuhani, Albaraa M Aati, Zahra H Al Ghazwi, Raed S Alruwaili

**Affiliations:** 1 Neurosurgery, King Fahad Central Hospital, Abu Arish, SAU; 2 College of Medicine, University of Hail, Hail, SAU; 3 College of Medicine, Alexandria University, Alexandria, EGY; 4 College of Medicine, King Faisal University, Al-Ahsa, SAU; 5 College of Medicine, Jazan University, Jazan, SAU; 6 College of Medicine, King Saud University, Riyadh, SAU; 7 Faculty of Medicine, University of Tabuk, Tabuk, SAU

**Keywords:** lumbar lordosis, pelvic incidence, pelvic tilt, preoperative planning, robotic-assisted spine surgery, sagittal balance, sagittal vertical axis, spinal alignment, spinal deformity, surgical planning accuracy

## Abstract

Preoperative surgical planning is central to achieving optimal spinal alignment in spinal deformity (SD) surgery; however, the extent to which different planning modalities accurately translate preoperative goals into surgical outcomes remains incompletely characterized. This systematic review evaluates the accuracy and clinical usefulness of existing preoperative planning tools in SD surgery. A systematic search of MEDLINE, Embase, and the Cochrane Library was conducted in accordance with the Preferred Reporting Items for Systematic Reviews and Meta-Analyses (PRISMA) guidelines. Studies reporting quantitative accuracy data for any preoperative planning modality in SD surgery were included. Study quality was assessed using the Newcastle-Ottawa Scale, the National Institutes of Health (NIH) Quality Assessment Tool, and the Cochrane Risk of Bias 2 (RoB 2) tool for randomized controlled trials. A total of 32 studies involving more than 3,500 patients published between 2006 and 2025 were included, encompassing the following six planning modalities: robotic-assisted navigation, three-dimensional (3D)-printed patient-specific guides, virtual surgical planning (VSP) software, advanced imaging techniques, mathematical regression formulas, and artificial intelligence (AI)-assisted platforms. Pedicle screw placement demonstrated high rates of accuracy overall with planning-assisted techniques than with 72-90% with free-hand techniques. A limited proportion of cases achieved simultaneous attainment of all three primary sagittal alignment targets, despite per-parameter achievement rates exceeding 68%. The integration of patient-specific rods within virtual surgical planning (VSP) contributed to improved alignment outcomes. Three-dimensional printing-guided osteotomy demonstrated a substantial reduction in neurological complications compared with traditional freehand techniques. Planning-assisted technologies improve surgical accuracy across multiple modalities in SD surgery; however, simultaneous achievement of all sagittal correction goals remains challenging. Key limitations include the lack of standardized outcome measures and the need for prospective comparative studies evaluating the relationship between planning accuracy and patient-reported outcomes. This systematic review aimed to map available planning tools, evaluate their accuracy in achieving preoperative goals, and identify factors associated with planning success or failure.

## Introduction and background

Spinal deformity (SD) refers to a heterogeneous group of spinal alignment disorders in adults, with a prevalence of up to 68% in individuals over 60 years of age [1-5]. SD is strongly associated with back pain, functional disability, and reduced quality of life [6-10].

Surgical treatment of SD remains one of the most complex procedures in orthopedic and neurosurgical practice, with reported complication rates of 39-71% and revision rates exceeding 25% at five years [3,5,8]. Successful outcomes depend on accurately translating preoperative alignment goals into intraoperative correction. Failure to achieve target spinopelvic parameters, particularly pelvic incidence minus lumbar lordosis (PI-LL mismatch), sagittal vertical axis (SVA), and pelvic tilt (PT), is associated with persistent disability, mechanical failure, and proximal junctional kyphosis [11-17].

In recent years, several preoperative planning modalities have been developed to improve surgical accuracy in SD. These include robotic-assisted navigation systems, three-dimensional (3D) printed patient-specific models and guides, virtual surgical planning (VSP) software, advanced intraoperative imaging techniques, and biomechanical or regression-based planning models [15-20]. These approaches differ in their mechanisms, ranging from intraoperative robotic guidance to patient-specific 3D-printed templates for osteotomy planning and implant positioning.

Despite increasing adoption of these technologies, comparative evidence regarding their planning accuracy remains limited and fragmented due to heterogeneity in study design, outcome definitions, and patient populations [21-32]. No prior systematic review has comprehensively synthesized accuracy outcomes across all major SD preoperative planning modalities.

## Review

Methodology

This systematic review was conducted in accordance with the Preferred Reporting Items for Systematic Reviews and Meta-Analyses (PRISMA) guidelines [33].

Literature Search and Screening

A systematic search of MEDLINE, Embase, and the Cochrane Library was performed for studies published between January 2000 and March 2025. The search strategy combined terms related to spinal disorders and surgery with preoperative planning concepts, including variations of spinal deformity, degenerative spinal disease, and surgical planning tools. No language restrictions were applied during the search; however, only English-language full-text articles were included. Reference lists of eligible studies were manually screened to identify additional relevant publications. Table 1 outlines the comprehensive search strategy used to identify studies evaluating preoperative planning technologies in SD surgery. The search combined controlled vocabulary and free-text terms related to spinal deformity and preoperative planning modalities, including robotic-assisted systems, three-dimensional printing, virtual surgical planning, artificial intelligence, and regression-based models. Filters were applied to include human studies published in English with available full-text articles. Additional studies were identified through manual screening of the reference lists of eligible articles.

**Table 1:** Electronic database search strategy for identification of eligible studies.

Database	Search terms/strategy	Time frame	Filters applied
MEDLINE (via PubMed)	(“spinal deformity” OR scoliosis OR kyphosis OR “degenerative spine”) AND (“preoperative planning” OR “surgical planning” OR “robotic surgery” OR “navigation” OR “3D printing” OR “patient-specific guide” OR “virtual surgical planning” OR “AI” OR “machine learning” OR “regression model” OR “spinopelvic parameters”)	January 2000-March 2025	English language, human studies, full-text articles
Embase	(‘spinal deformity’ OR scoliosis OR kyphosis OR ‘degenerative spine’) AND (‘preoperative planning’ OR ‘surgical planning’ OR ‘robot-assisted surgery’ OR navigation OR ‘3D printing’ OR ‘patient-specific guide’ OR ‘virtual surgical planning’ OR AI OR ‘machine learning’ OR ‘predictive model’)	January 2000-March 2025	English language, human studies
Cochrane Library	(“spinal deformity” OR scoliosis OR kyphosis) AND (“preoperative planning” OR “surgical planning” OR robotics OR navigation OR “3D printing” OR “virtual planning”)	January 2000-March 2025	Trials and reviews, English language
Manual Search	Reference lists of included studies and relevant reviews	N/A	Studies meeting the inclusion criteria

Eligibility Criteria

Studies were selected using the Population, Intervention, Comparison, and Outcomes (PICO) framework [34]. The population included patients undergoing surgery for SD, including adult scoliosis, kyphosis, and degenerative spinal conditions such as degenerative disc disease, spondylolisthesis, and spondylosis affecting the cervical, thoracic, or lumbar spine. Table 2 shows the PICO framework used to define eligibility criteria for study inclusion in this systematic review. The population included adults undergoing surgery for spinal deformity and related degenerative spinal conditions. Interventions comprised a range of preoperative planning technologies, including robotic-assisted navigation, 3D-printed patient-specific guides, virtual surgical planning, artificial intelligence-based systems, regression-based predictive models, and advanced imaging techniques. Comparators included conventional planning approaches or direct comparisons between planned and achieved surgical outcomes. Outcomes focused on quantitative measures of planning accuracy, including pedicle screw placement accuracy, spinal alignment correction parameters, osteotomy precision, complication rates, and predictive performance of planning models.

**Table 2:** PICO framework for study selection criteria. SVA: sagittal vertical axis; PT: pelvic tilt; PI-LL mismatch: pelvic incidence minus lumbar lordosis; DTI: diffusion tensor imaging; T1PA: T1 pelvic angle

Component	Description
Population (P)	Patients undergoing surgery for adult spinal deformity (ASD), including scoliosis, kyphosis, and degenerative spinal conditions (e.g., degenerative disc disease, spondylolisthesis, spondylosis)
Intervention (I)	Preoperative planning technologies, including robotic-assisted navigation, 3D-printed patient-specific guides/models, virtual surgical planning (VSP), artificial intelligence (AI)-based systems, regression-based alignment formulas, and advanced imaging modalities (CT, MRI, CT myelography, DTI)
Comparison (C)	Conventional planning methods (e.g., free-hand techniques, standard radiographic planning) or comparison between planned and actual surgical outcomes
Outcomes (O)	Quantitative measures of planning accuracy, including pedicle screw placement accuracy, Cobb angle correction, sagittal and coronal alignment parameters (PI-LL mismatch, SVA, PT, T1PA), osteotomy accuracy, achievement of alignment targets, complication rates (e.g., rod fracture, proximal junctional kyphosis), and predictive accuracy of planning models

Interventions included any preoperative planning method, such as robotic-assisted navigation systems, 3D-printed patient-specific models or guides, VSP software, regression-based alignment prediction models, advanced imaging techniques (computed tomography {CT}, magnetic resonance imaging {MRI}, CT myelography, diffusion tensor imaging), and AI-based planning systems. Comparators included either conventional planning without advanced tools or direct comparison between planned and executed surgical outcomes.

Outcomes required at least one quantitative measure of planning accuracy, including pedicle screw placement accuracy, Cobb angle correction, thoracic kyphosis or lumbar lordosis restoration, sagittal and coronal alignment parameters (PI-LL mismatch, SVA, PT, T1 pelvic angle), osteotomy angle accuracy, achievement of planned correction goals, rod fracture, or proximal junctional kyphosis (PJK). Studies were excluded if they lacked quantitative outcome data or were conference abstracts, editorials, narrative reviews, or animal studies.

Data Extraction

Two independent reviewers extracted data using a standardized form and cross-verified all entries. Extracted variables included author, year, country, study design, sample size, patient demographics, clinical indication, surgical procedure, planning modality, outcome measures, follow-up duration, and key findings. Disagreements were resolved by discussion or adjudication by a senior reviewer.

Quality and Risk of Bias Assessment

Study quality was assessed using validated tools according to study design. The Newcastle-Ottawa Scale (NOS) was applied to comparative observational studies to assess selection, comparability, and outcomes [35]. The National Institutes of Health (NIH) quality assessment tool for before-and-after studies without a control group was used for single-arm studies and case series [36]. Randomized controlled trials (RCTs) were assessed using the Cochrane Risk of Bias tool version 2 (RoB 2). All assessments were performed independently by two reviewers, with disagreements resolved by a third reviewer [37].

Data Synthesis

Due to substantial heterogeneity in study design, patient populations, interventions, and outcome definitions, a meta-analysis was not performed. Findings were synthesized narratively and structured according to planning modality and outcome domain. Where comparable numerical data were available, descriptive statistics and reported ranges were summarized. Primary outcome domains included pedicle screw placement accuracy, sagittal alignment correction, osteotomy accuracy, and prediction of alignment targets using planning models.

Results

Search Results and Study Selection

**Figure 1:**
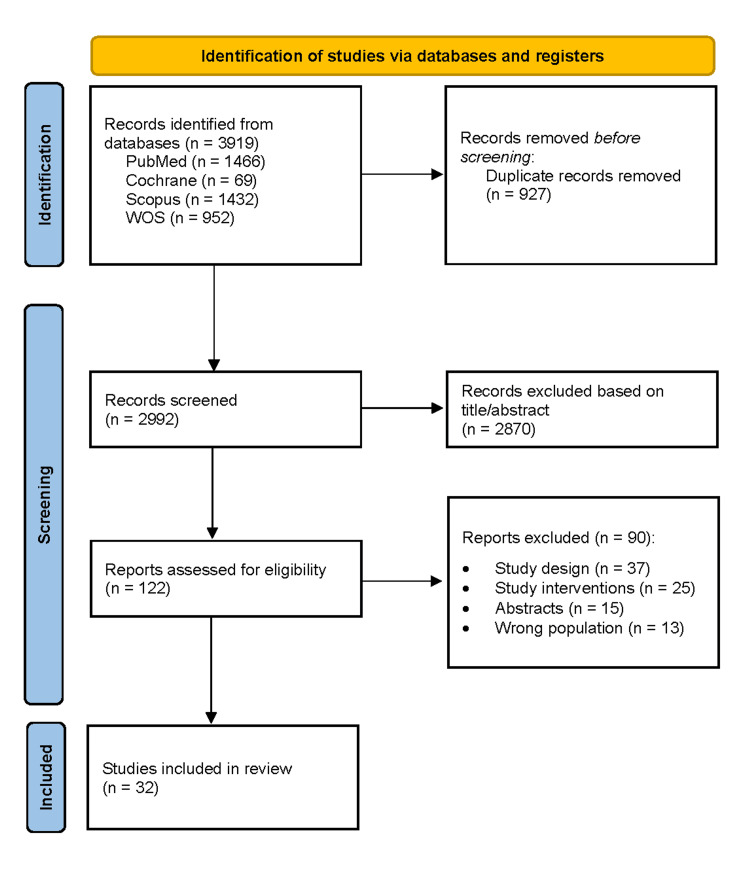
PRISMA flow diagram depicting the study selection process for the systematic review. Preferred Reporting Items for Systematic Reviews and Meta-Analyses (PRISMA) flow diagram detailing the study selection process for this systematic review [33]. Following the identification of records through database searching and other sources, duplicates were removed, and the remaining records were screened. Full-text articles were assessed for eligibility, with exclusions documented along with reasons. Studies meeting the inclusion criteria were included in the final synthesis.

A systematic search identified 2,992 records after deduplication. Following title and abstract screening, 122 full-text articles were assessed for eligibility. A total of 32 studies met the inclusion criteria and were included in the qualitative synthesis (Figure 1) [1-32].

Characteristics of Included Studies

A total of 32 included studies were published between 2006 and 2025 and included over 3,500 patients (Tables 3-5). Most were retrospective observational studies (n=17), followed by prospective cohort studies (n=3), prospective clinical studies (n=3), randomized controlled trials (RCTs; n=2), a prospective validation study (n=1), a prospective case series (n=1), and other single-arm case series (n=5). Most studies were single-center, with limited multicenter data.

**Table 3:** Characteristics of included studies evaluating preoperative planning modalities in spinal deformity surgery. This table summarizes the key characteristics of the 32 studies included in the systematic review of preoperative planning technologies in SD surgery. Data are presented for study identification, country, study design, recruitment period, sample size, surgical procedure, surgical technique, preoperative planning modality, comparator (when applicable), inclusion criteria, and principal findings. The included studies evaluate a range of planning approaches, including robotic-assisted navigation systems, three-dimensional (3D) printed patient-specific guides and models, VSP software, regression-based and formula-driven alignment prediction models, artificial intelligence (AI)-based planning systems, advanced imaging modalities (CT, MRI, CTM, and DTI), and virtual reality (VR)-based planning systems. Missing or unavailable data not explicitly reported in the original studies are indicated as “not reported” (NR). SD: spinal deformity; ACDF: anterior cervical discectomy and fusion; CTM: CT myelography; DTI: diffusion tensor imaging; LL: lumbar lordosis; MIS: minimally invasive surgery; PLIF: posterior lumbar interbody fusion; PSO: pedicle subtraction osteotomy; PT: pelvic tilt; SVA: sagittal vertical axis; TLIF: transforaminal lumbar interbody fusion; VCR: vertebral column resection; VSP: virtual surgical planning; VR: virtual reality; TK: thoracic kyphosis; ASD: adult spinal deformity; T1PA: T1 pelvic angle; AIS: adolescent idiopathic scoliosis; GAP: global alignment and proportion

Studies	Country	Study design	Recruitment period	Sample size	Procedure	Surgical technique	Planning method	Comparator	Inclusion criteria	Key findings
Archavlis et al. (2018) [1]	Germany	Retrospective observational	January 2012-January 2016	194	Single-level lumbar fusion with posterior pedicle screw instrumentation (TLIF/PLIF L1-S1)	(1) Percutaneous robot-assisted instrumentation, (2) percutaneous fluoroscopy-guided instrumentation, (3) open midline pedicle screw insertion	Robot group: CT-based 3D planning (axial/sagittal/coronal) using the Renaissance system {Memphis, TN: Mazor Robotics}; other groups: CT-based biplanar planning with manual angle measurement	-	Degenerative spondylolisthetic stenosis (grade I-II)	Careful preoperative planning of the entry point reduces the risk of superior facet joint violation
Barzilay et al. (2006) [2]	Israel	Prospective cohort	March 2005-November 2005	15	Spinal fusion procedures	SpineAssist guidance system (Memphis, TN: Mazor Robotics)	CT-based 3D spine model planning using system-generated reconstruction	-	Patients undergoing spinal fusion	The robotic system enables highly accurate screw placement but has a steep learning curve
Benech et al. (2020) [3]	Italy	Retrospective observational	NR	52	Posterolateral lumbosacral fusion	Minimally invasive navigated robotic guidance	CT-based preoperative robotic trajectory planning transferred to the navigation system	-	Lumbosacral pedicle screw fixation cases	Robotic navigation showed acceptable deviation from planned trajectories
Berjano et al. (2014) [4]	Spain	Retrospective observational	January 2008-January 2014	50	PSO	PSO	PI- and age-based non-geometric formulas for LL and TK planning	-	Sagittal imbalance requiring PSO	Age/PI-based model (method Bis) is more accurate than the regression formula
Cecchinato et al. (2019) [5]	Italy	Randomized controlled trial	January 2015-October 2016	29	Spinal deformity correction with pedicle screws	Posterior instrumentation	3D-printed patient-specific guides (MySpine)	Free-hand technique	ASD (idiopathic, congenital, degenerative deformity; surgical indication; consent)	CT-based guides improve accuracy and safety vs. free-hand placement
Cool et al. (2021) [6]	The Netherlands	Retrospective observational	February 2020-September 2020	5	Posterior spinal pedicle screw instrumentation	Guided pedicle screw placement	CT-based 3D planning with patient-specific 3D-printed guides	-	Thoracolumbar deformity surgery patients	3D guides are safe and improve screw accuracy
Jia et al. (2023) [7]	China	Prospective cohort	June 2022-December 2022	45	Thoracolumbar fixation surgery	Internal fixation	AI-based deep learning (3D U-Net; Freiburg, Germany: University of Freiburg) CT planning system	Free-hand placement	Lumbar degenerative disease or kyphosis	AI planning improves safety and feasibility vs. conventional planning
Kisinde et al. (2021) [8]	USA	Prospective cohort	2017-2019	33	Spinal deformity correction	Mazor X Align robotic system	CT-based robotic 3D planning integrated with radiographic parameters	-	ASD correction surgery patients	Preoperative robotic planning reliably predicts alignment outcomes
Lafage et al. (2022) [9]	USA	Retrospective observational	2006-2009	99	Single-level lumbar PSO	PSO	Regression-based spinopelvic alignment formulas (PI, LL, TK, age)	-	ASD patients undergoing single-level lumbar PSO	Formulas validated for predicting postoperative alignment
Lafage et al. (2012) [10]	USA	Retrospective observational	NR	433	ASD corrective realignment	Posterior spinal fusion	Software-based regression modeling (SpineView; Fremont, CA: SpineView, Inc.)	-	ASD patients with radiographic deformity criteria	LL correction depends on the level of correction and affects global alignment
Lee et al. (2012) [11]	South Korea	Prospective validation study	NR	20	Lumbar fusion with pedicle screws	Standard pedicle screw fixation	CT-based automated trajectory optimization model	Manual surgeon planning	Lumbar degenerative disease	Automated planning improves screw safety and reduces breaches
Li et al. (2021) [12]	China	Randomized controlled trial	January 2018-October 2018	56	Posterior lumbar interbody fusion	Robot-assisted vs. free-hand	CT-based robotic 3D planning of screw trajectory	Weinstein free-hand technique	Lumbar stenosis with sciatica/claudication	Robotic system improves accuracy and adherence to plan
McCarthy et al. (2024) [13]	USA	Retrospective observational	2009-2018	1048	ASD corrective surgery	Posterior spinal fusion with osteotomies	Regression-based LL prediction model (PI, TK, age, T1PA)	-	ASD adults meeting radiographic deformity criteria	The formula improves alignment targeting in surgical planning
Oe et al. (2022) [14]	China	Retrospective observational	2010-2016	203	ASD corrective surgery	Posterior fusion	Hamamatsu formula+GAP/Roussouly classification systems	-	Adults aged ≥18 years, had ≥5-year follow-up, underwent ≥4 levels fusion	Alignment guided by Roussouly improves mechanical outcomes
Park et al. (2014) [15]	South Korea	Retrospective observational	2007-2010	18	Kyphotic deformity correction	PSO	Computer simulation (Surgimap-based osteotomy planning; New York City, NY: Globus Medical)	-	Ankylosing spondylitis kyphosis	Preoperative simulation improves surgical planning accuracy
Passias et al. (2019) [16]	USA	Retrospective observational	2016-2017	34	Cervical deformity surgery	Posterior cervical fusion	Surgimap simulation+patient-specific rod planning	No planning/standard rods	Cervical deformity with radiographic criteria	Planning improves postoperative alignment correction
Prost et al. (2020) [17]	France	Prospective clinical study	NR	77	Posterior vertebral fixation	PSO and posterior bone resection	Patient-specific rod design using software simulation	-	ASD, AIS, Parkinson-related deformity	Patient-specific rods feasible with good short-term outcomes
Schöller et al. (2020) [18]	Germany	Retrospective observational	2011-2012	20	Cervical decompression	ACDF/corpectomy/laminectomy	MRI, CT myelography, diffusion tensor imaging	-	Cervical spondylotic myelopathy	DTI may replace CTM for surgical planning
Sharma et al. (2018) [19]	India	Retrospective observational	2008-2015	138	Lumbar fusion	Posterior fusion	MRI vs. standing radiographs for flexibility assessment	-	ASD with sagittal imbalance	MRI better predicts flexible deformity correction
Shiban et al. (2016) [20]	Germany	Prospective clinical study	NR	26	Lumbar decompression	Microsurgical decompression	MR myelography-based planning	Conventional myelography	Lumbar degenerative disease	MR myelography alters surgical indication decisions
Tan et al. (2018) [21]	USA	Retrospective observational	2015-2016	23	Spinal deformity correction	Pedicle screw fixation	3D-printed spine model-assisted planning	Free-hand technique	Complex spinal deformity	3D models improve safety in severe deformities
Thayaparan et al. (2020) [22]	Australia	Prospective clinical study	2016-2018	129	MIS TLIF	Minimally invasive fusion	CT-based patient-specific surgical planning software	-	MIS TLIF candidates	Patient-specific planning improves operative efficiency
Tsai et al. (2017) [23]	Taiwan	Retrospective observational	2013-2015	125	Transpedicular fixation	Robotic-assisted screw placement	CT-based Renaissance robotic planning system	-	Degenerative lumbar disease	Accurate planning essential for robotic screw placement
van Dijk et al. (2015) [24]	Netherlands	Retrospective observational	NR	112	MIS lumbar fusion	SpineAssist robotic guidance	CT-based robotic trajectory planning	-	Robotic PLIF patients	The robotic system enables the accurate execution of the planned trajectory
Volk et al. (2023) [25]	Germany	Retrospective observational	2018-2020	122	Pedicle screw fixation	Open/MIS robot-assisted	CT-based navigation (Mazor system; Memphis, TN: Medtronic)	-	Robotic spinal fusion patients	High screw accuracy with objective deviation measurement
Weinberg et al. (2025) [26]	USA	Retrospective observational	2015-2023	146	ASD correction	Long-segment fusion	Virtual surgical planning+machine learning rod design	Conventional planning	ASD long-segment fusion with ≥1-year follow-up	VSP improves radiographic accuracy and reduces rod fracture
Yamato et al. (2016) [27]	Japan	Retrospective observational	2013	116	Sagittal realignment surgery	Thoracolumbar fusion	Regression model for LL and PT prediction	-	ASD corrective surgery	Equation-based LL target improves alignment planning
Alsofy et al. (2021) [28]	Germany	Retrospective observational	2010-2017	73	Cervical decompression	Ventral/dorsal foraminotomy	3D VR reconstruction from CT	-	Cervical foraminal stenosis	VR improves surgical planning and strategy selection
Alsofy et al. (2019) [29]	Germany	Retrospective observational	2007-2018	171	Spinal fusion surgery	MIS or open fusion	3D VR reconstruction (CT/MRI-based)	-	Degenerative instability with failed conservative treatment	VR influences surgical decision-making strategy
Zhang et al. (2012) [30]	China	Prospective case series	NR	3	Pedicle guidewire placement	Robot-assisted screw placement	CT-based 3D reconstruction planning	-	Patients requiring pedicle fixation	Spine Bull’s-Eye robot (Beijing, China: Beijing Jishuitan Hospital) enables accurate screw placement
Zhang et al. (2024) [31]	China	Retrospective observational/matched comparative cohort	2020-2023	40	Three-column osteotomy	PSO/VCR	3D simulation+patient-specific printed guides	Simulation-only planning	Severe spinal deformity (>80° Cobb or rigid deformity)	3D guides improve accuracy and safety
Smith et al. (2024) [32]	USA	Prospective cohort	NR	266	Long posterior spinal fusion	Posterior and/or anterior approach	Surgical planning software (Surgimap, GAP, Roussouly)	No standardized planning	Severe ASD radiographic deformity	Achieving alignment targets remains inconsistent

**Table 4:** Baseline characteristics of study populations included in spinal deformity surgical planning studies. This table summarizes baseline demographic and clinical characteristics of patients included in studies evaluating preoperative planning methods in spinal deformity (SD) surgery and related spinal conditions. Data are presented by study, including planning method, sample size, sex distribution (male, n {%}), age (years, mean {standard deviation; SD}), body mass index (BMI, kg/m², mean {SD}), and underlying spinal condition(s). When multiple intervention arms were reported within a study, each cohort is listed separately. The table includes studies evaluating robotic-assisted systems, three-dimensional (3D)-printed patient-specific guides, artificial intelligence (AI)-based planning software, VSP, imaging-based planning (including CT, MRI, and CT myelography-based approaches), regression- and formula-based alignment prediction models, and virtual reality (VR)-based planning systems. Missing or unavailable data not explicitly reported in the original studies are indicated as “not reported” (NR). AIS: adolescent idiopathic scoliosis; ASD: adult spinal deformity; LL: lumbar lordosis; VSP: virtual surgical planning; VR: virtual reality

Studies	Planning method	Sample size	Male, n (%)	Age, years mean (SD)	BMI, kg/m² mean (SD)	Condition
Archavlis et al. (2018) [1]	Robotic percutaneous	58	22 (38)	51 (12)	26 (5)	Spondylolisthetic stenosis and degenerative disc disease
	Fluoroscopic percutaneous	64	30 (46.8)	53 (11)	24 (4)	Same
	Open midline	72	32 (44.4)	49 (13)	23 (7)	Same
Barzilay et al. (2006) [2]	SpineAssist guidance system (Memphis, TN: Mazor Robotics)	15	NR	NR	NR	Degenerative spine disorders
Benech et al. (2020) [3]	Navigated robotic guidance	52	37 (71.2)	49.8 (11.3)	25.5 (4)	Degenerative disc disease, spondylolisthesis
Berjano et al. (2014) [4]	Non-geometrical calculation rules	50	NR	65 (8)	NR	Adult spinal deformity
Cecchinato et al. (2019) [5]	3D-printed patient-specific guide	14	2 (14.3)	34 (15.3)	NR	AIS, adult degenerative scoliosis, congenital deformity
	Free-hand	15	1 (6.7)	26 (17.2)	NR	Same
Cool et al. (2021) [6]	3D-printed guide	5	2 (40)	18.2 (5.4)	NR	Idiopathic/syndromic/neuromuscular scoliosis, kyphosis
Jia et al. (2023) [7]	AI-based planning software	45	15 (33.3)	67.6 (8.3)	26.1 (3.5)	Lumbar degenerative disease, thoracic kyphosis
Kisinde et al. (2021) [8]	Robotic planning (Mazor X Align)	33	7 (21.2)	51 (23)	23.9 (4.2)	Mixed spinal deformity
Lafage et al. (2022) [9]	Spinopelvic formulas	99	19 (19.2)	55.5 (11.7)	NR	Adult spinal deformity
Lafage et al. (2012) [10]	SpineView regression software	433	81 (18.7)	62.9 (9.7)	28.1 (5.5)	Adult spinal deformity
Lee et al. (2012) [11]	CT-based automated planning	20	8 (40)	53 (12.5)	NR	Degenerative disc disease, stenosis
Li et al. (2021) [12]	Robotic system (Orthbot; Shenzhen, China: Shenzhen FUTURTEC Medical Technology Co., Ltd.)	27	13 (48.1)	48.9 (10.1)	24.2 (NR)	Lumbar canal stenosis
	Free-hand	29	16 (55.2)	50.8 (12.6)	24.7 (NR)	Same
McCarthy et al. (2024) [13]	Regression formula	1048	271 (25.8)	60.7 (14.3)	28.1 (6.2)	Adult spinal deformity
Oe et al. (2022) [14]	Preoperative planning strategies	203	29 (14.3)	63.5 (16.0)	22.8 (3.9)	Adult spinal deformity
Park et al. (2014) [15]	Surgimap simulation (New York City, NY: Globus Medical)	18	17 (94.4)	36.5 (NR)	NR	Ankylosing spondylitis kyphosis
Passias et al. (2019) [16]	Planning+patient-specific rods	15	14 (41.2)	57.4 (8.0)	29.6 (7.7)	Adult spinal deformity
	No planning, standard rods	19	NR	NR	NR	Same
Prost et al. (2020) [17]	Patient-specific rod planning	77	22 (29)	59.1 (18.6)	NR	ASD, AIS, Parkinson deformity
Schöller et al. (2020) [18]	Imaging-based planning	20	11 (55)	60.8 (7.5)	NR	Cervical spondylotic myelopathy
Sharma et al. (2018) [19]	MRI-based planning	138	42 (30)	61 (NR)	NR	Lumbar sagittal imbalance
Shiban et al. (2016) [20]	MR/myelography-based planning	26	13 (50)	NR	NR	Complex lumbar degenerative disease
Tan et al. (2018) [21]	3D-printed spine model	23	10 (43.5)	35.7 (NR)	NR	Complex spinal deformity
	Free-hand	20	3 (15)	NR	NR	Same
Thayaparan et al. (2020) [22]	Patient-specific 3D BioModels	129	54 (41.9)	59.2 (13.1)	NR	Degenerative spinal disease
Tsai et al. (2017) [23]	Renaissance robotic guidance	125	38 (30.4)	65.7 (12.6)	25.5 (3.4)	Degenerative lumbar disease
van Dijk et al. (2015) [24]	Robot-guided screw placement	112	67 (59.8)	56.8 (12.5)	NR	Spinal instability/deformity
Volk et al. (2023) [25]	Mazor navigation system	122	50 (41.0)	62 (12)	30 (5.6)	Mixed degenerative conditions
Weinberg et al. (2025) [26]	Virtual surgical planning	61	33 (54)	62.1 (10.6)	32.4 (9.2)	Adult spinal deformity
	Traditional planning	85	41 (48)	64.3 (7.9)	31.2 (6.5)	Same
Yamato et al. (2016) [27]	Mathematical LL formula	116	NR	66 (NR)	NR	Adult spinal deformity
Alsofy et al. (2021) [28]	VR-based planning	171	91 (53.2)	54.7 (11.9)	NR	Lumbar degenerative disease
Alsofy et al. (2019) [29]	VR-based planning	73	NR	NR	NR	Cervical foraminal stenosis
Zhang et al. (2024) [31]	Patient-specific template	20	12 (60)	36.7 (12.4)	23.7 (2.0)	Adult spinal deformity
	Non-template group	20	12 (60)	36.3 (11.8)	23.6 (1.9)	Same
Zhang et al. (2012) [30]	Spine Bull’s-Eye robot (Beijing, China: Beijing Jishuitan Hospital)	3	1 (33.3)	60.3 (19.3)	NR	Lumbar spondylolisthesis, meningomyelocele
Smith et al. (2024) [32]	Surgical planning tools	266	85 (32)	61 (14.6)	27.5 (5.8)	Adult spinal deformity

**Table 5:** Outcomes and accuracy of preoperative planning interventions in spinal deformity surgery. This table summarizes reported outcomes and accuracy metrics from studies evaluating preoperative planning interventions in SD surgery. Interventions include robotic-assisted planning systems, three-dimensional (3D)-printed patient-specific guides, AI-based planning software, VSP, regression-based and spinopelvic predictive formulas, imaging-based planning (including CT, MRI, CTM, and DTI), VR-based planning systems, and computer-assisted simulation tools. Outcomes include pedicle screw placement accuracy (reported using Gertzbein-Robbins grading or equivalent classification systems), facet joint violation, spinal alignment accuracy (including SVA, PT, PI-LL, and TK), osteotomy accuracy, predictive performance of alignment models, and influence on surgical decision-making or strategy selection. Comparators, when available, include free-hand techniques, fluoroscopy-guided surgery, conventional planning, or simulation-only planning. ASD: adult spinal deformity; CTM: CT myelography; DTI: diffusion tensor imaging; GR: Gertzbein-Robbins; ICC: intraclass correlation coefficient; LL: lumbar lordosis; NPV: negative predictive value; PI: pelvic incidence; PI-LL: pelvic incidence minus lumbar lordosis mismatch; PSAV: pedicle standard axis view; PT: pelvic tilt; PPV: positive predictive value; SVA: sagittal vertical axis; TK: thoracic kyphosis; T1PA: T1 pelvic angle; VR: virtual reality; VSP: virtual surgical planning; SD: spinal deformity; T1PA: T1 pelvic angle

Studies	Planning strategy	Comparator	Outcome measure	Result
Archavlis et al. (2018) [1]	Robot-assisted planning	Fluoroscopy/open surgery	Facet joint violation	97% vs. 78% vs. 94%
Barzilay et al. (2006) [2]	SpineAssist robotic planning	None	CT-confirmed screw accuracy	40% successful cases
Benech et al. (2020) [3]	CT-based robotic planning	None	Gertzbein-Robbins grade A/B	0.983
Berjano et al. (2014) [4]	Regression formula (method A)	PI+10 rule (method B)	Predictive accuracy of sagittal balance	Method B showed lower prediction error
Cecchinato et al. (2019) [5]	3D-printed patient-specific guides	Free-hand technique	Safe screw placement (Gertzbein grades 0/A)	90.2% vs. 83.1%
Cool et al. (2021) [6]	3D-printed guides	None	Safe screw placement (Gertzbein grades 0/A)	1
Jia et al. (2023) [7]	AI-based planning (Surgiplan)	Free-hand	Gertzbein-Robbins grade A accuracy	85.1% vs. 64.9%
Kisinde et al. (2021) [8]	Robotic planning (Mazor X Align)	None	Planned vs. achieved spinal alignment	100% screws in safe zone
Lafage et al. (2022) [9]	Spinopelvic regression formulas	None	Prediction of SVA and PT	PPV 76-84%, NPV 98%
Lafage et al. (2012) [10]	Software-based radiographic mapping with regression formulas	None	Predictive sagittal balance modeling	1° L4-S1 LL change → 1° T1PA, 10 mm SVA, 0.5° PT change
Lee et al. (2012) [11]	Automated CT-based planning	Manual planning	Planning accuracy and safety margin	100% success; improved safety margin
Li et al. (2021) [12]	Orthbot robotic planning (Shenzhen, China: Shenzhen FUTURTEC Medical Technology Co., Ltd.)	Free-hand	Screw accuracy and facet violation	Similar accuracy; lower facet violation
McCarthy et al. (2024) [13]	Regression formulas	Age-integrated formula	Prediction of lumbar lordosis	Mean error 1.3° vs. 0.4°
Oe et al. (2022) [14]	Hamamatsu/GAP/Roussouly methods	Comparative evaluation	Mechanical complications	Roussouly restoration reduced complications
Park et al. (2014) [15]	Surgimap computer simulation (New York City, NY: Globus Medical)	None	Predicted vs. achieved alignment	Strong correlation (ICC 0.6-0.9)
Passias et al. (2019) [16]	Preoperative planning+patient-specific rods	Standard rods	Cervical sagittal alignment	Improved alignment outcomes
Prost et al. (2020) [17]	Patient-specific rods	None	Alignment correction (SVA, PI-LL)	SVA -41%, PI-LL -62% improvement
Schöller et al. (2020) [18]	CTM+DTI-based planning	None	Planned vs. achieved decompression	All planned decompressions performed as indicated
Sharma et al. (2018) [19]	Supine MRI-based LL estimation	Full-length scoliosis radiographs	Prediction of LL and PI-LL mismatch	Superior predictive accuracy
Shiban et al. (2016) [20]	MR myelography	Conventional myelography/postmyelography CT	Surgical planning accuracy	Comparable accuracy; good interobserver agreement
Tan et al. (2018) [21]	3D-printed spine model	Free-hand	Screw accuracy (Gertzbein-Robbins grade A/B)	96.3% vs. 96.3%
Thayaparan et al. (2020) [22]	Patient-specific 3D-printed kits	None	Screw accuracy (Gertzbein-Robbins grade A/B)	0.978
Tsai et al. (2017) [23]	Renaissance robotic planning	None	Screw accuracy grading	98.5% grade I
van Dijk et al. (2015) [24]	SpineAssist robotic planning	None	Screw placement accuracy (Gertzbein-Robbins grade A/B)	0.979
Volk et al. (2023) [25]	Mazor navigation planning	None	Screw placement accuracy (Gertzbein-Robbins grade A/B)	0.972
Weinberg et al. (2025) [26]	Virtual surgical planning software	Conventional planning	Spinopelvic parameter accuracy	73.8% SVA accuracy; 72.1% T1PA achievement
Alsofy et al. (2019) [29]	3D VR reconstruction (CT-based)	None	Influence on surgical decision-making	Influenced ventral approach strategy; no effect on dorsal strategy
Yamato et al. (2016) [27]	Lumbar lordosis predictive formula	None	Prediction of pelvic tilt	Strong correlation (r=0.82)
Alsofy et al. (2021) [28]	3D VR reconstruction (CT/MRI-based)	None	Influence on surgical decision-making	Significantly influenced therapy choice
Zhang et al. (2024) [31]	3D simulation+printed osteotomy guide	Simulation only	Osteotomy angle match	89-90% vs. 74-80%
Zhang et al. (2012) [30]	Robot-assisted PSAV technique	None	PSAV acquisition and guidewire insertion success	1
Smith et al. (2024) [32]	Surgical planning tools	None	Achievement of alignment targets	~70% per parameter (SVA 74.4%, PI-LL 71.4%, TK 68.8%)

Study populations primarily included SD, degenerative lumbar disease, sagittal imbalance, spinal stenosis, and scoliosis. Surgical procedures most commonly involved posterior spinal fusion, pedicle screw instrumentation, pedicle subtraction osteotomy (PSO), and minimally invasive transforaminal lumbar interbody fusion (MIS-TLIF). Six categories of preoperative planning modalities were identified as follows: robotic-assisted systems (eight studies), 3D-printed guides or models (six studies), regression-based or spinopelvic prediction formulas (four studies), advanced imaging-based planning using CT, MRI, CT myelography, or diffusion tensor imaging (five studies), radiographic or simulation software (four studies), and patient-specific rod or VSP systems (three studies). One study evaluated an AI-based pedicle screw planning system using deep learning.

Most studies compared advanced planning tools with conventional techniques, including free-hand screw placement, standard radiographic planning, or conventional rod contouring. Geographically, studies were conducted in Europe (n=11), Asia (n=9), North America (n=5), and Australia (n=1), reflecting broad international adoption of preoperative planning technologies in spine surgery.

Risk of Bias and Quality Assessment

A total of 12 comparative observational studies were assessed using the Newcastle-Ottawa Scale (NOS). Overall, five studies were rated low risk and seven high risk. Common limitations included poor cohort comparability, incomplete baseline reporting, and limited follow-up duration.

A total of 17 single-arm studies were assessed using the National Institutes of Health (NIH) quality assessment tool for before-and-after studies without control groups. Sixteen studies (94%) were rated as good quality and one (6%) as fair quality. Common limitations included a lack of blinded outcome assessment and inadequate control of confounding variables. Two RCTs were assessed using the Cochrane Risk of Bias tool version 2 (RoB 2). Overall, studies demonstrated low to moderate risk of bias, with the most common concerns related to blinding and outcome assessment (Tables 6-8).

**Table 6:** Risk of bias assessment of randomized controlled trials using the Cochrane RoB 2 tool. *Overall judgment downgraded due to “some concerns” in one domain. Risk of bias for randomized controlled trials was assessed using the Cochrane Risk of Bias 2 (RoB 2) tool [37]. Domains included as follows: (D1) bias arising from the randomization process; (D2) bias due to deviations from intended interventions; (D3) bias due to missing outcome data; (D4) bias in measurement of the outcome; and (D5) bias in selection of the reported result.

Studies	Domain 1	Domain 2	Domain 3	Domain 4	Domain 5	Overall risk of bias
Cecchinato et al. (2019) [5]	Low	Low	Low	Low	Low	Low
Li et al. (2021) [12]	Low	Low	Low	Low	Some concerns	Low*

**Table 7:** Quality assessment of observational comparative studies using the NOS. Study quality for observational comparative studies was assessed using the Newcastle-Ottawa Scale (NOS), which evaluates the following three domains: selection (maximum four stars), comparability (maximum two stars), and outcome (maximum three stars) [35]. Asterisks (*) indicate awarded points. Studies were categorized as good or poor quality based on overall star allocation and methodological rigor. Hyphen (-) indicates data not reported or not applicable.

Studies	Representativeness	Selection of non-exposed	Exposure ascertainment	Outcome absent at baseline	Comparability	Outcome assessment	Follow-up duration	Follow-up adequacy	Quality
Archavlis et al. (2018) [1]	*	*	*	*	*	*	*	*	Good quality
Berjano et al. (2014) [4]	*	*	*	*	-	*	*	*	Poor quality
Jia et al. (2023) [7]	*	*	*	*	*	*	*	*	Good quality
Lee et al. (2012) [11]	-	-	*	*	-	*	*	*	Poor quality
McCarthy et al. (2024) [13]	*	*	*	*	*	*	*	*	Good quality
Oe et al. (2022) [14]	*	*	*	*	**	*	*	*	Good quality
Passias et al. (2019) [16]	-	*	*	*	-	*	*	*	Poor quality
Sharma et al. (2018) [19]	*	*	*	*	-	*	*	*	Poor quality
Shiban et al. (2016) [20]	-	-	*	*	-	*	*	*	Poor quality
Tan et al. (2018) [21]	*	-	*	*	*	*	*	*	Good quality
Weinberg et al. (2025) [26]	*	*	*	*	**	*	*	*	Good quality
Zhang et al. (2024) [31]	*	*	*	*	**	*	*	*	Good quality

**Table 8:** Quality assessment of non-comparative studies using the NIH quality assessment tool for before-and-after and case series studies. Study quality for non-comparative studies was assessed using the National Institutes of Health (NIH) quality assessment tool for before-and-after studies and case series [36]. Items Q1-Q14 correspond to predefined methodological criteria evaluating study design, population, exposure, and outcome assessment, follow-up, and confounding. Responses include yes, no, not reported (NR), and not applicable (NA). Final ratings were categorized as good, fair, or poor quality based on overall methodological rigor. Q1. Was the study question or objective clearly stated? Q2. Was the study population clearly specified and defined? Q3. Was the participation rate of eligible persons at least 50%? Q4. Were all subjects selected or recruited from the same or similar populations (including the same time period)? Were inclusion and exclusion criteria prespecified and applied uniformly? Q5. Was a sample size justification, power description, or variance and effect estimate provided? Q6. Were the exposure(s) of interest measured prior to the outcome(s)? Q7. Was the timeframe sufficient to reasonably expect an association between exposure and outcome? Q8. For variable exposures, were different levels of exposure examined (e.g., categories or continuous measures)? Q9. Were exposure measures clearly defined, valid, reliable, and consistently implemented? Q10. Was the exposure assessed more than once over time? Q11. Were outcome measures clearly defined, valid, reliable, and consistently implemented? Q12. Were outcome assessors blinded to exposure status? Q13. Was the loss to follow-up after baseline ≤20%? Q14. Were key potential confounding variables measured and adjusted for statistically?

Studies	Q1	Q2	Q3	Q4	Q5	Q6	Q7	Q8	Q9	Q10	Q11	Q12	Q13	Q14	Quality
Barzilay et al. (2006) [2]	Yes	Yes	Yes	Yes	NR	NA	Yes	NA	Yes	NA	Yes	NR	Yes	No	Good quality
Benech et al. (2020) [3]	Yes	Yes	Yes	Yes	NR	NA	Yes	NA	Yes	NA	Yes	Yes	Yes	No	Good quality
Cool et al. (2021) [6]	Yes	Yes	Yes	Yes	NR	NA	Yes	NA	Yes	NA	Yes	NR	Yes	No	Good quality
Kisinde et al. (2021) [8]	Yes	Yes	Yes	Yes	NR	NA	Yes	NA	Yes	NA	Yes	NR	Yes	No	Good quality
Lafage et al. (2022) [9]	Yes	Yes	Yes	Yes	NR	NA	Yes	NA	Yes	NA	Yes	NR	Yes	No	Good quality
Lafage et al. (2012) [10]	Yes	Yes	Yes	Yes	NR	NA	Yes	NA	Yes	NA	Yes	NR	Yes	No	Good quality
Park et al. (2014) [15]	Yes	Yes	Yes	Yes	NR	NA	Yes	NA	Yes	NA	Yes	NR	Yes	No	Good quality
Prost et al. (2020) [17]	Yes	Yes	Yes	Yes	NR	NA	Yes	NA	Yes	NA	Yes	NR	Yes	No	Good quality
Schöller et al. (2020) [18]	Yes	Yes	Yes	Yes	NR	NA	Yes	NA	Yes	NA	Yes	Yes	Yes	No	Good quality
Smith et al. (2024) [32]	Yes	Yes	Yes	Yes	NR	NA	Yes	NA	Yes	NA	Yes	NR	Yes	Yes	Good quality
Thayaparan et al. (2020) [22]	Yes	Yes	Yes	Yes	NR	NA	Yes	NA	Yes	NA	Yes	NR	Yes	NR	Good quality
Tsai et al. (2017) [23]	Yes	Yes	Yes	Yes	NR	NA	Yes	NA	Yes	NA	Yes	NR	Yes	Yes	Good quality
van Dijk et al. (2015) [24]	Yes	Yes	Yes	Yes	NR	NA	Yes	NA	Yes	NA	Yes	NR	Yes	No	Good quality
Volk et al. (2023) [25]	Yes	Yes	Yes	Yes	NR	NA	Yes	NA	Yes	NA	Yes	Yes	Yes	No	Good quality
Yamato et al. (2016) [27]	Yes	Yes	Yes	Yes	NR	NA	Yes	NA	Yes	NA	Yes	NR	Yes	No	Good quality
Alsofy et al. (2019) [29]	Yes	Yes	Yes	Yes	NR	NA	Yes	NA	Yes	NA	Yes	NR	Yes	No	Good quality
Alsofy et al. (2021) [28]	Yes	Yes	Yes	Yes	NR	NA	Yes	NA	Yes	NA	Yes	NR	Yes	No	Good quality
Zhang et al. (2012) [30]	Yes	No	NA	Yes	NR	NA	Yes	NA	Yes	NA	Yes	NR	Yes	No	Fair quality

Pedicle Screw Placement Accuracy

A total of 14 studies reported pedicle screw placement accuracy. Planning-assisted techniques achieved accuracy rates of 96.3-100%, compared with 72-90% for free-hand techniques. Robotic-assisted systems demonstrated the highest accuracy, including 100% accuracy reported by Barzilay et al. [2], 98.5% by Kisinde et al. [8], and 97.7% by van Dijk et al., with minimal high-grade breaches [24]. Li et al. reported significantly lower cortical breach rates with robotic guidance compared with free-hand placement (3.7% vs. 17.4%) [12]. Overall, planning-assisted systems consistently improved screw placement accuracy across platforms.

Sagittal Alignment Goal Achievement

Five studies evaluated the achievement of preoperative sagittal alignment targets. In the largest prospective multicenter study (n=266), Smith et al. reported achievement rates of 74.4% for SVA, 71.4% for PI-LL mismatch, and 68.8% for thoracic kyphosis (TK), with simultaneous achievement of all targets in 37.2% of patients [32]. Factors associated with success included use of picture archiving and communication system (PACS)-based planning, lower global coronal angle, and avoidance of three-column osteotomy.

Weinberg et al. evaluated VSP systems with or without patient-specific rods and found superior age-adjusted T1 pelvic angle (T1PA) correction in the planning group (72.1% vs. 30.6%, p<0.001) [26]. Rod fracture rates were also lower in the planning group, although patient-reported outcomes were similar at one year.

Osteotomy Planning Accuracy

Zhang et al. compared 3D-printed patient-specific guides with free-hand three-column osteotomy in 40 patients. The guided group demonstrated higher coronal (89.9% vs. 74.5%) and sagittal (90.5% vs. 80.4%) accuracy, reduced neurological complications (5% vs. 35%), and shorter operative time by approximately 40 min [31]. These findings support improved precision and safety with patient-specific 3D guidance in complex osteotomies.

Mathematical Regression Formulas

Five studies evaluated regression-based formulas for predicting lumbar lordosis (LL). McCarthy et al. developed an age-adjusted formula (LL = PI - 0.3TK - 0.5Age + 10), with a mean absolute error of 5.0° [13]. However, only 32.9% of patients achieved age-adjusted alignment targets postoperatively, indicating a gap between predicted and achieved outcomes. Other formulas, including those by Lafage et al. and Yamato et al., improved population-level prediction accuracy when incorporating pelvic incidence (PI) and age, but none demonstrated consistent improvements in clinical outcomes when used alone [10,14].

AI-assisted and Advanced Imaging Planning

Jia et al. evaluated an AI-based planning platform and reported clinically acceptable alignment targets in 91.3% of cases vs. 74.2% with conventional planning (p=0.04) [7]. Studies assessing computer-assisted radiographic planning showed improved rod contouring and implant positioning; however, consistent improvements in patient-reported outcomes were not demonstrated across studies [15,29].

Limitations

This review is limited by the predominance of observational studies, which restrict causal inference regarding the superiority of specific preoperative planning modalities. Substantial heterogeneity in outcome definitions (e.g., Gertzbein-Robbins grading, angular deviation, match ratios, and binary goal achievement) prevented quantitative synthesis and limited direct comparability across studies. In addition, follow-up durations varied widely, and few studies assessed long-term maintenance of alignment or patient-reported outcomes in relation to planning accuracy, limiting conclusions on durable clinical impact.

## Conclusions

Planning-assisted technologies appear to improve the accuracy of SD surgery across different modalities. However, achieving all predefined sagittal alignment objectives simultaneously remains challenging. Future research should focus on standardizing outcome measures and conducting prospective comparative studies to better clarify the relationship between planning accuracy and clinically meaningful patient outcomes.
